# Ultra-Tough Polylactide/Bromobutyl Rubber-Based Ionomer Blends *via* Reactive Blending Strategy

**DOI:** 10.3389/fchem.2022.923174

**Published:** 2022-06-16

**Authors:** Xingfang Zhang, Xu Lu, Dong Huang, Yingli Ding, Jinshan Li, Zhenyu Dai, Liming Sun, Jin Li, Xiaohui Wei, Jie Wei, Yang Li, Kunyu Zhang

**Affiliations:** ^1^ School of Chemical Engineering and Technology, Tianjin University, Tianjin, China; ^2^ Petrochemical Research Institute, PetroChina Company Limited, Beijing, China; ^3^ School of Materials Science and Engineering, Tianjin University, Tianjin, China

**Keywords:** polylactic acid, bromobutyl rubber, ionomers, reactive blending, toughening

## Abstract

A series of ultra-toughened sustainable blends were prepared from poly(lactic acid) (PLA) and bromobutyl rubber-based ionomers (*i*-BIIRs) *via* reactive blending, in which dicumyl peroxide (DCP) and Joncryl®ADR-4440 (ADR) were used as reactive blending additives. The miscibility, phase morphology and mechanical property of the PLA/*i*-BIIRs blends were thoroughly investigated through DMA, SEM, tensile and impact tests. The influence of different ionic groups and the effects of DCP and ADR on the compatibility between the phases, phase structure and mechanical properties were analyzed. The introduction of the imidazolium-based ionic groups and the reactive agents enable the *i*-BIIRs play multiple roles as effective compatibilizers and toughening agents, leading to improved interfacial compatibility and high toughness of the blends. The mechanical properties test showed that the PLA/*i*-BIIRs blends exhibit excellent toughness: impact strength and the elongation at break of AR-OH(30)+AD reached 95 kJ/m^2^ and 286%, respectively. The impact fracture surface showed the large-scale plastic deformation of the PLA matrix in the blends, resulting in greatly absorbing the impact energy. The results proved that simultaneously applying reactive blend and multiple intermolecular interactions methods is an effective toughening strategy for toughening modification of the PLA blends.

## Graphical Abstract

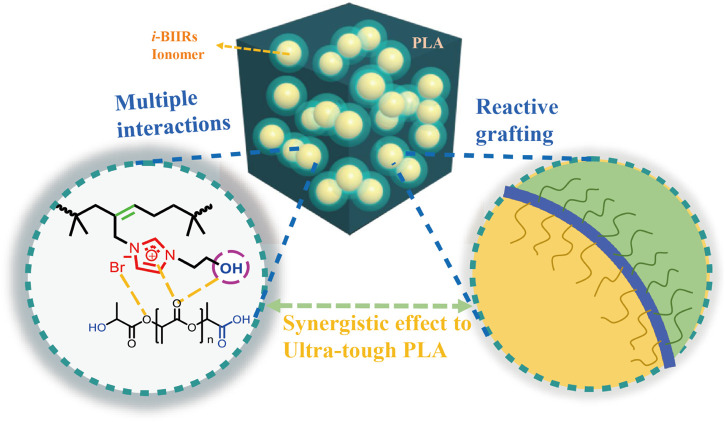

## 1 Introduction

Poly(lactic acid) (PLA) is a kind of bio-based polyester obtained by chemical synthesis from lactic acid, which is widely derived from renewable resources such as corn starch. PLA has very good biodegradability and biocompatibility (Farah et al., 2016; Nazrin et al., 2020). Moreover, the high tensile strength, good transparency and excellent processing properties make it have the potential to replace petroleum-based polymers in many fields (Farah et al., 2016; Nagarajan et al., 2016; Dubey et al., 2017). The wide application of high-performance PLA-based materials is of great significance to solve the white pollution and energy crisis (Luo et al., 2020). However, the poor toughness of PLA seriously limits the large-scale market application. Therefore, toughening modification of PLA has become a research hotspot in academia and industry (Kfoury et al., 2013).

Among the toughening modification methods, melt blending is widely used in the modification of various brittle polymers due to the advantages of economic efficiency and easy scale (Sangeetha et al., 2018; Zhao et al., 2020). By melt blending, different polymers with complementary properties can be quickly and massively prepared into multi-component blends with excellent comprehensive properties. Blending PLA with flexible elastomers to obtain high-performance materials has proven to be an effective means of PLA toughening (Zhang et al., 2014; Wu et al., 2019). However, the poor compatibility between most elastomers and the PLA matrix is the principal obstacle to obtaining desired performance of PLA-based materials (Zhao et al., 2020). Currently, the main compatibilization strategies for PLA blends are adding block or graft copolymer compatibilizers, reactive blending compatibilization, and introducing intermolecular interactions between the components (Lins et al., 2015; Rosli et al., 2019; Jiang et al., 2020; Ding et al., 2021). The preparation of copolymers often requires complex technological processes, which is not conducive to the large-scale application of blend materials. As for the method of reactive blending and introduction of intermolecular interaction, the compatibilization effect is limited by the lack of active functional groups in the chain structure of PLA, resulting in an unsatisfying modification effect. Therefore, simply and efficiently improving the interfacial compatibility is the key challenge to prepare the tough PLA-based material for the large-scale application.

Recently, we developed a series of ionomers containing organic ionic groups as modifiers for PLA to effectively improve the performance of PLA (Chen et al., 2018; Huang et al., 2020; Li et al., 2020; Sun et al., 2020). The imidazolium ion group has excellent structural designability, endowing the ionomers with plentiful functionalities and good performance. Through the design of the imidazolium group, functional groups such as hydroxyl and amino groups can be easily introduced, which can promote the formation of multiple intermolecular interactions between components. In addition, the multiple intermolecular interactions caused by the functional groups make the imidazolium-based polymerized ionic liquids (PILs) have a variety of unique properties (Cui et al., 2017; Nie et al., 2019). More importantly, these functional groups from the imidazolium group will also provide active sites for the next reaction modification. On this basis, we synthesized a series of imidazole-based poly(isobutylene-co-isoprene) ionomers (*i*-BIIRs) for the toughening modification of PLA (Huang et al., 2020).

In this work, we used the previously synthesized bromobutyl rubber ionomer with hydroxyl-functionalized imidazolium cationic structure to synergize with dicumyl peroxide (DCP) and Joncryl^®^ ADR-4440 (ADR) as reaction assistants, and a series of PLA/*i*-BIIRs blends with excellent mechanical properties were prepared by the reactive blending. The toughening mechanism of the blends was further explored through the systematic characterization and analysis of the mechanical properties, compatibility and phase morphology of the blends. Benefiting from the double bond of *i*-BIIRs and the hydroxyl-functionalized imidazolium cationic group, the synergistic effect of *in situ* reaction compatibilization and non-valent interaction, the experimental results show that the introduction of the ionic group improves the compatibility between the two components and the blend system obtains good mechanical properties.

## 2 Experimental Section

### 2.1 Materials

PLA (Ingeo PLA 3001D, M_w_ = 1.7 × 10^5^ g/mol, a density of 1.25 g/cm^3^, melt index of 3.0 g/10 min) was purchased from NatureWorks LLC (United States). Bromobutyl rubber (BIIR, BB 2030, bromine content of 1.14 wt%, 3.18 mol% allylic bromide functionality/g BIIR) was purchased from Lanxess, Germany. N-(2-Hydroxyethyl)-imidazole was used as received from Acros Organics Ltd. 1-ethylimidazole (98%) was supplied by J&K Scientific Ltd. Dicumyl peroxide (DCP) was obtained from Shanghai Aladdin Biochemical Technology Co. Ltd. Joncryl®ADR-4440 (ADR) was purchased from BASF, Germany.

### 2.2 Synthesis of the Imidazolium-Functionalized Ionomers

**FIGURE 1:**
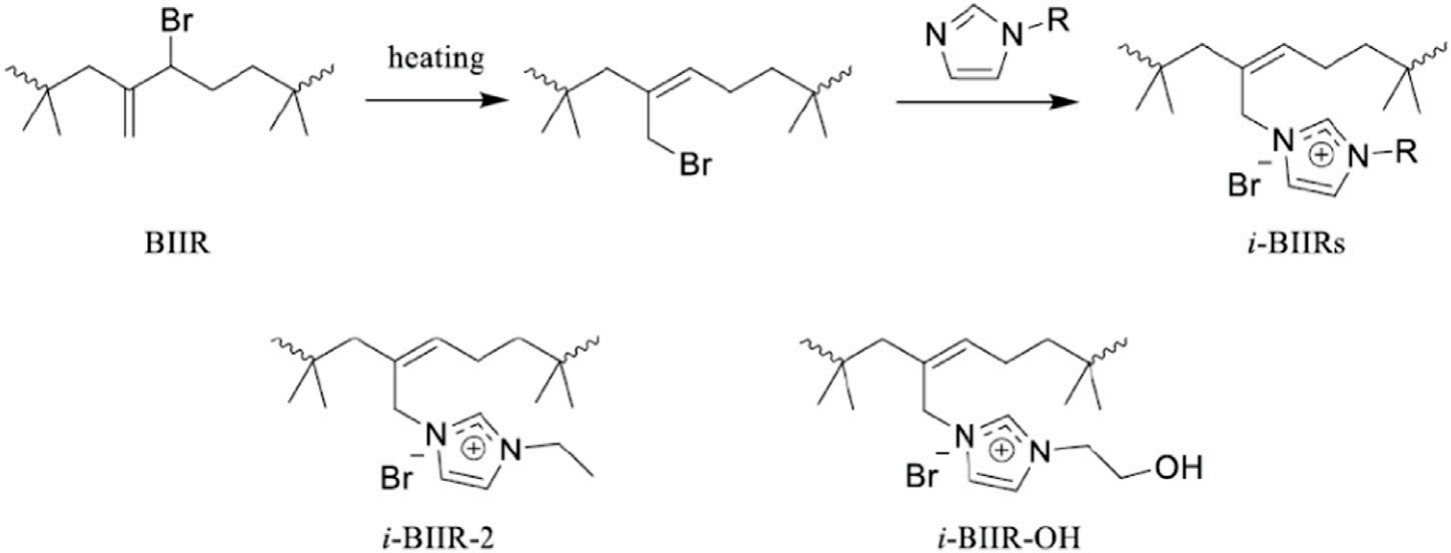
Schematic diagram of the ionization reaction of BIIR and the structure of the ionomers.

*i*-BIIRs were synthesized by previously reported methods (Huang et al., 2020). N-(2-Hydroxyethyl)-imidazole or 1-ethylimidazole reacted with BIIR in toluene solution at 110°C for 11 h. After cooled down to about room temperature, the reaction product was purified several times by precipitation in acetone. The products (*i*-BIIR-OH, *i*-BIIR-2) were obtained after drying in a vacuum oven at 50°C for 24 h. The corresponding reaction scheme is shown in Figure 1.

### 2.3 Preparation of the Blends

Before blending, PLA, BIIR and *i*-BIIRs were dried in a vacuum oven for 24 h at 60°C. The samples were reactively blended by a twin-screw microcompounder to obtain the PLA/*i*-BIIRs blends. All the blends were obtained after 5 min mixing process under the conditions of screw speed 60 rpm and temperature 180°C. Then, the tensile and impact strips of the samples were obtained by injection molding, the injection cylinder temperature was 175°C, the mold temperature was 50°C, and the pressure was maintained for 20 s. The neat PLA was also processed in the same way as the blends. The blend ratios of all blend samples are listed in Table 1.

**TABLE 1:** Sample compositions.

Samples	PLA (wt%)	BIIR (wt%)	*i*-BIIR-2 (wt%)	*i*-BIIR-OH (wt%)	DCP (wt%)	ADR (wt%)
AR(30)+AD	70	30	-	-	0.5	0.5
AR-2(30)+AD	70	-	30	-	0.5	0.5
AR-OH(30)	70	-	-	30	-	-
AR-OH(30)+A	70	-	-	30	-	0.5
AR-OH(30)+D	70	-	-	30	0.5	-
AR-OH(10)+AD	90	-	-	10	0.5	0.5
AR-OH(20)+AD	80	-	-	20	0.5	0.5
AR-OH(30)+AD	70	-	-	30	0.5	0.5
AR-OH(40)+AD	60	-	-	40	0.5	0.5

### 2.4 Testing and Characterization Methods

#### 2.4.1 Dynamic Mechanical Analysis Test

A DMA Q800 system (TA Instruments) was used to analyze the dynamic mechanical properties of the blends. The test adopted the single-cantilever mode, the test oscillating frequency was 1 Hz, and the amplitude is 15 μm. Then the data was tested from −85 to 125°C using a ramp rate of 3 C/min.

#### 2.4.2 Scanning Electron Microscopy Test

All of the samples were observed by an SEM system of Hitachi, Japan (s4800) for phase morphology. The cryofracture surface morphology was characterized separately by an SEM system (HITACHI S-4800). The samples were cryo-fractured under the freezing of liquid nitrogen and then dried in a vacuum oven at 40°C for 12 h to remove moisture. After sputter-coated with gold, the morphology of the samples was observed by SEM with a 5 kV voltage. Images of the fracture surfaces of the impact specimens were also observed by the SEM system with the same voltage and sputter-coated with gold.

#### 2.4.3 Tensile and Impact Test

The tensile properties of the samples were tested by a SANS tensile machine with a 1 kN load cell. The measurement was performed according to ASTM D638 (type I), and the tensile rate during the test was 10 mm/min. The impact strength of the samples was tested using an Izod impact testing machine, with a spline size of 80 mm × 12.5 mm × 4 mm and a notch depth of 2 mm, and the test results were the average of the test results of five splines.

#### 2.4.4 Torque Evolution

The evolution of torque during the reaction blending process of the PLA/*i*-BIIRs blends was measured by a HAAKE rheometer (HAAKE Rheomix 600, Germany). PLA and ADR, *i*-BIIRs, and DCP were added sequentially and mixed at a rotor speed of 60 rpm at 180°C.

## 3 Results and Discussion

### 3.1 Dynamic Mechanical Properties

**FIGURE 2:**
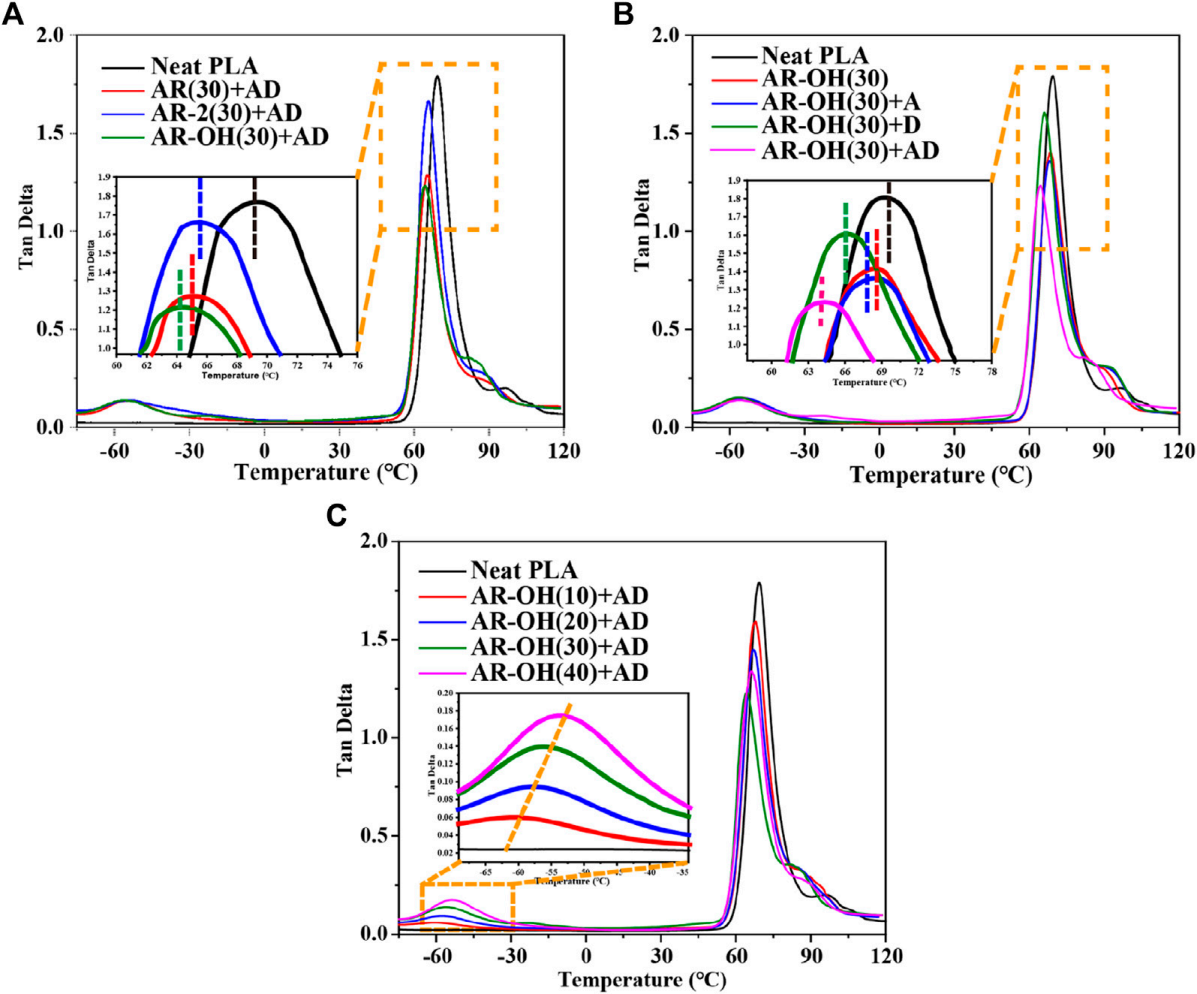
Tan δ curves of the PLA/*i*-BIIRs blends obtained by DMA: **(A)** neat PLA and PLA/*i*-BIIRs blends with ADR and DCP added and 30 wt% content of ionomer, **(B)** neat PLA and PLA/*i*-BIIR-OH blends with different addition of ADR and DCP, **(C)** neat PLA and PLA/*i*-BIIR-OH blends with different ionomer content.

In toughened polymer blends, the compatibility between the components directly affects the mechanical properties of the blends. Figure 2 shows the dynamic mechanical curves of PLA and the PLA/*i*-BIIRs blends. The effect of reactive blending on the compatibility between PLA and the ionomer can be studied by analyzing the dynamic relaxation transition behavior of the components. Figure 2A firstly compares the effects of different ionomers on the dynamic mechanical behavior of the blend components under the combination of reaction assistants. Compared with the pure PLA, the tanδ peak of the PLA phase in the blends shifted to low temperature, which indicates that the compatibility between the components was significantly improved under the conditions of the reactive blending. It is noted that the *i*-BIIR-OH ionomer shows the best compatibilization effect among the toughening agents. Therefore, we selected AR-OH(30) blend as the basic formulation for the next step of research on the influence of the reactive DCP and ADR additives. As shown in Figure 2B, the combinations of ADR and DCP additives show more remarkable compatibilization effects for the blends compared to that of the blend with single additive added. In the case of the coexistence of ADR and DCP, the glass transition temperature of the PLA phase in the system obviously shifts to a lower temperature compared to that of the other blends when ADR and DCP act alone. In addition, we also try to obtain the optimum formulation with desirable mechanical properties by changing component content. Figure 2C shows the effect of increasing *i*-BIIR-OH content on the dynamic mechanical behavior of the blend system. With the increase of the ionomer content, the glass transition relaxation peak of PLA is further approached to lower temperature, and the relaxation transition peak corresponding to *i*-BIIR-OH also moves to high temperature at the same time. This further indicates that the increase in ionomer content is beneficial to the improvement of blend compatibility.

**SCHEME 1:**
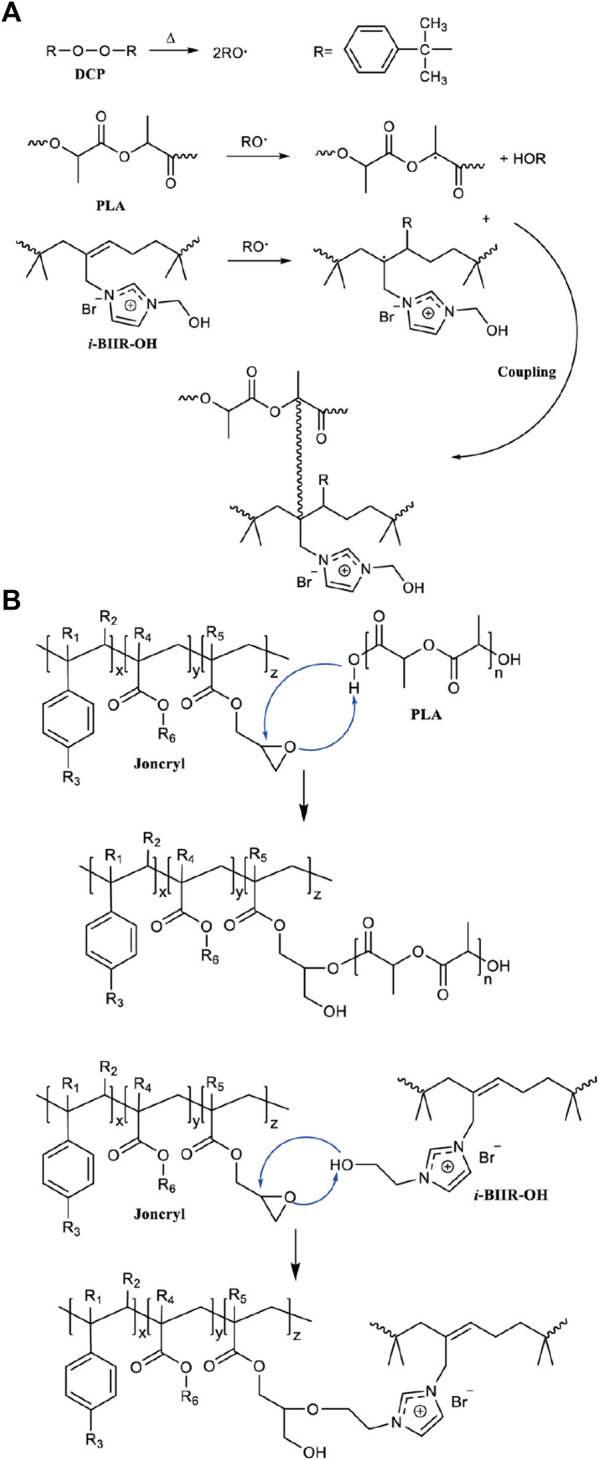
Schematic diagram of the main reaction during the reactive blending: **(A)** the reaction caused by the addition of DCP, **(B)** the reaction caused by the addition of ADR.

**FIGURE 3:**
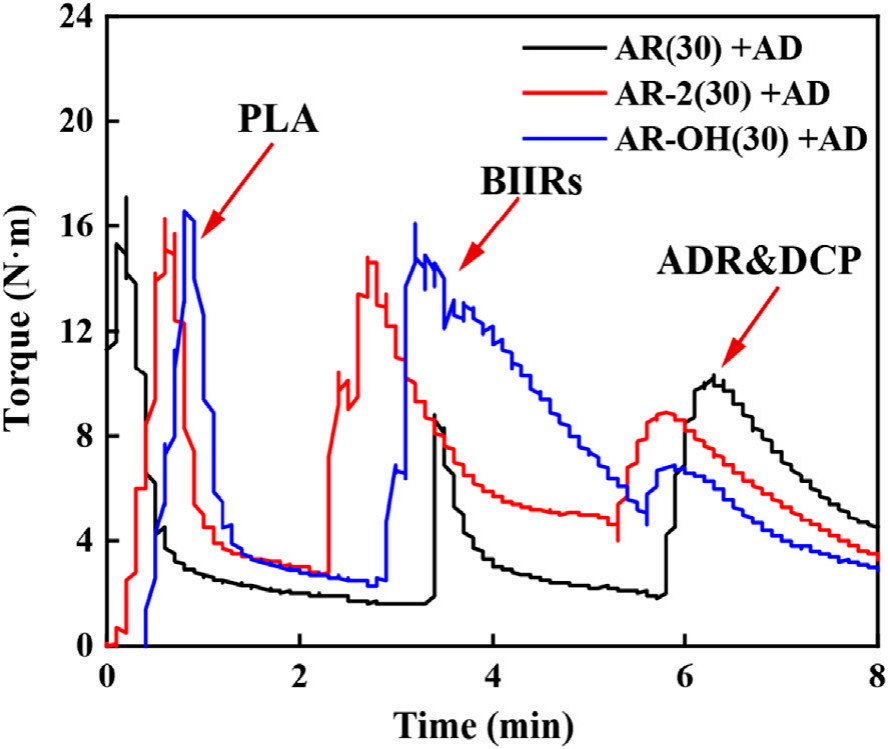
Torque curves during melt mixing process of the PLA/*i*-BIIRs blends.

The DMA results show that the coexistence of ionomers containing -OH functional groups and the two reactive additives is the key factor to obtain the optimal compatibilization effect. This is mainly attributed to the multifunctional groups of the *i*-BIIR-OH ionomer to achieve multiple synergistic compatibilization effects in the reactive blending system. First, as described in previous studies, the ionic groups and hydroxyl groups of *i*-BIIR-OH can form multiple non-covalent interactions with the ester group and the end group of PLA(Lin et al., 2007; Livi et al., 2015; Megevand et al., 2016; Wang et al., 2021). Second, the double bond and hydroxyl functional groups of *i*-BIIR-OH can play as the active sites. The main reactions in the reactive blending process are shown in Scheme 1. During the reactive blending process, DCP is thermally decomposed into free radicals, which then react with the tertiary carbon atoms in PLA and the double bonds in the *i*-BIIR-OH ionomer to form macromolecular free radicals. Subsequently, these macromolecular radicals are coupled to form the copolymers of the two polymers (Si et al., 2018; Cao et al., 2020). Similarly, the epoxy group in ADR can react with the hydroxyl group in i-BIIR-OH and the carboxyl group and hydroxyl group in PLA, which creates covalent bonds between the two polymers (Yu et al., 2019; Farias da Silva and Soares, 2021). The backbone of ADR contains multiple epoxy groups, so the ADR molecules distributed in the phase interface region will eventually form a graft copolymer containing both the PLA chain and i-BIIR-OH chain. These copolymers formed during the reactive blending processes act as real compatibilizers to improve the compatibility of the blends. The above reaction compatibilization mechanism can be confirmed by the rotor torque curve with time during the blending process (Figure 3). Under the condition of high-temperature melt blending, the torque of the blending system increases obviously when ADR and DCP are added. The sudden increase in melt viscosity proves that the chemical reaction took place during the blending process, which can contribute to interfacial reaction compatibilization. At the same time, the influence of *i*-BIIR-OH content on the compatibilization effect of the blend system also proves the above mechanism. Increasing the ionomer content is beneficial to increasing the contact area of the two phases during the blending process, which promotes the *in-situ* grafting reaction and chain extension reaction between the interfaces, thereby improving the compatibility between the components.

### 3.2 Phase Morphology

**FIGURE 4:**
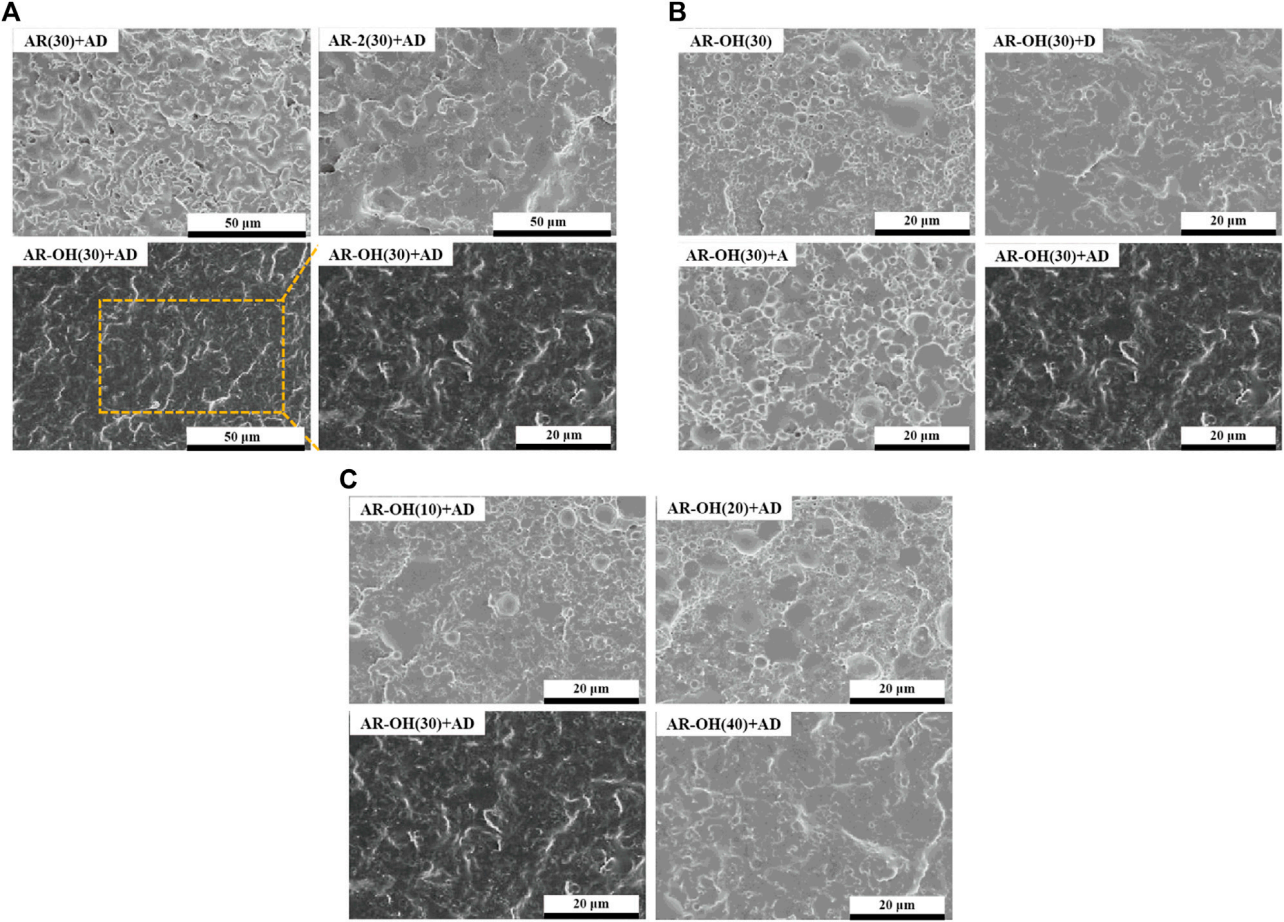
SEM micrographs of the cryofractured surfaces of the PLA/*i*-BIIRs blends. **(A)** neat PLA and PLA/*i*-BIIRs blends with ADR and DCP added and 30 wt% content of ionomer, **(B)** neat PLA and PLA/*i*-BIIR-OH blends with different addition of ADR and DCP, **(C)** neat PLA and PLA/*i*-BIIR-OH blends with different ionomer content.

The compatibility between the components of the blend has a decisive influence on the phase morphology of the blend, which greatly determines the mechanical properties of the polymer blend. Figure 4 shows the SEM morphologies of cryofractured surfaces of different PLA/*i*-BIIRs blends. It can be seen in Figure 4A that there is a relatively clear phase interface between the PLA matrix and the BIIR dispersed phase in the AR(30)+AD sample, indicating the limited compatibility of the blend. In contrast, the phase interface between the ionomers and the PLA matrix is blurred, indicating that the introduction of ionic groups is beneficial to improving the compatibility of PLA and the ionomers. This is consistent with our previously reported results (Huang et al., 2020). Compared with AR(30)+AD blend and AR-2(30)+AD, the phase interface of the AR-OH(30)+AD blend samples became more blurred, and dispersed particles of the ionomer phase were smaller and more uniform in size. This indicates that the ionomers containing -OH groups are beneficial to enhancing the compatibility of the blends.

Figure 4B compares the effect of different reactive additives on the phase structure of AR-OH(30) blends. As for the pristine AR-OH(30) blend, we note that large *i*-BIIR-OH particles uneven distribute in the PLA matrix with relatively clear interfaces. After single adding DCP or ADR into the blend, the interface between the phases become rougher than that of the pristine AR-OH(30) blend. Compared to ADR, DCP shows a better compatibilizing effect in the blend. The low amount of end functional groups in the PLA chains limited the efficiency of ADR as a coupling agent. However, the chain extension effect of ADR can increase the melt strength of PLA during the reactive blending process, which is beneficial to the dispersion of ionomers in the PLA matrix (Corre et al., 2011). Interestingly, when simultaneously adding ADR and DCP into the blend, the compatibility of the blends was remarkably improved owing to the *in-situ* grafting reaction at the interface and coupling reaction among the chains during reactive blending. As a result, the interfacial adhesion between the phases was significantly enhanced and no obvious phase-separation was observed for the AR-OH(30)+AD blend. Moreover, when the content of the *i*-BIIR-OH phase increases (Figure 4C), a more blurred phase interface can be observed, which is consistent with the previous DMA test results.

### 3.3 Mechanical Properties

**FIGURE 5:**
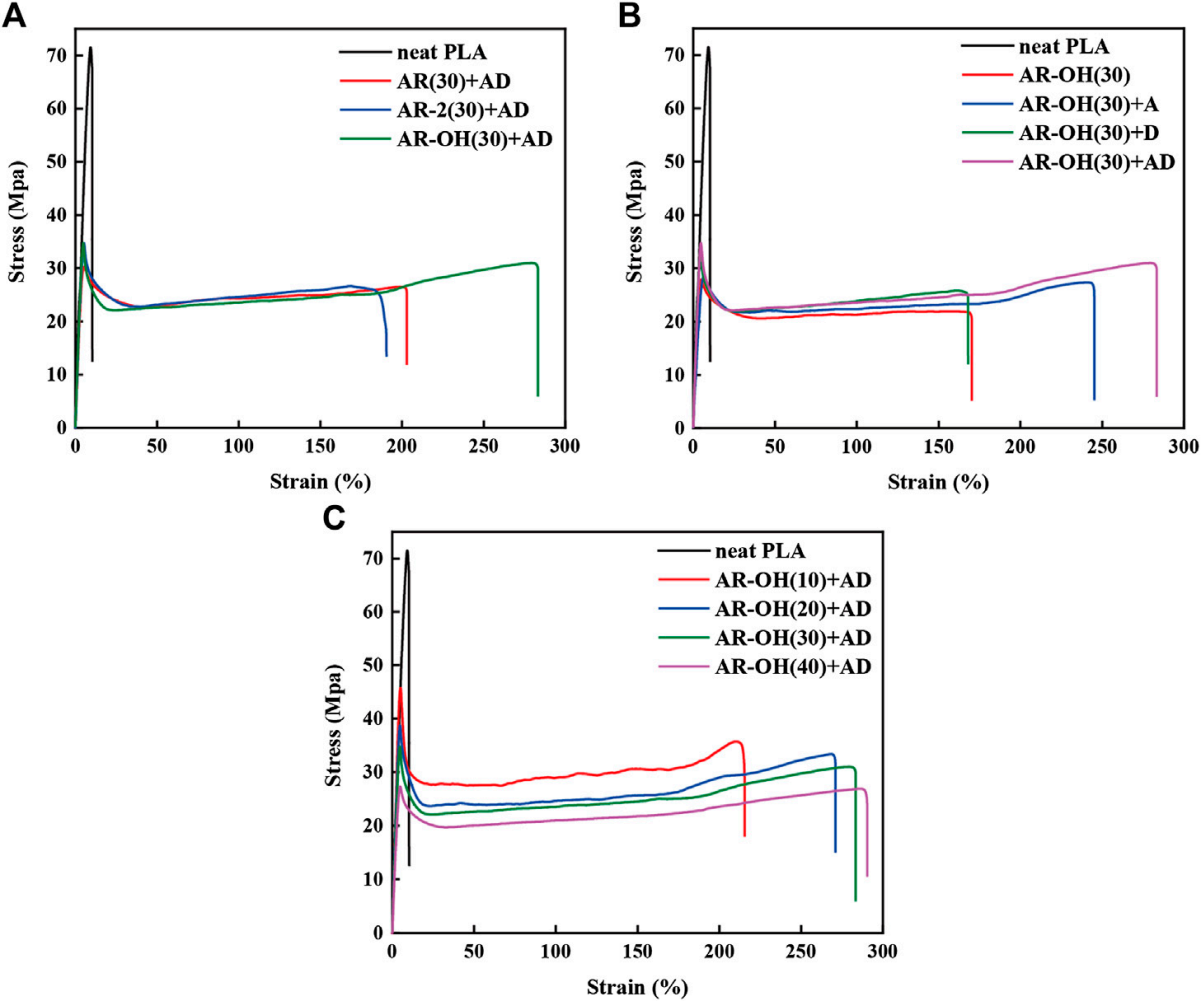
Tensile stress-strain curves of the PLA/*i*-BIIRs blends: **(A)** neat PLA and PLA/*i*-BIIRs blends with ADR and DCP added and 30 wt% content of ionomer, **(B)** neat PLA and PLA/*i*-BIIR-OH blends with different addition of ADR and DCP, **(C)** neat PLA and PLA/*i*-BIIR-OH blends with different ionomer content.

Figure 5 presents the stress-strain curves of different blends. The neat PLA shows poor flexibility with a very low elongation below 10%. Compared with the neat PLA, the elongation at break of the PLA/*i*-BIIRs blends was significantly improved to around 200%. Compared with AR(30)+AD and AR-2(30)+AD, AR-OH(30)+AD exhibited higher elongation at break of 286%. More interestingly, the tensile strength of the AR-OH(30)+AD blend was also increased (Figure 5A), which is seldom reported for toughening blend. It indicates that the presence of hydroxyl groups in the ionomer is beneficial to the improvement of the tensile properties of the blends. As mentioned above, the presence of hydroxyl groups plays an important role in improving the compatibility between BIIR and PLA. The improved interfacial adhesion will help the stress transfer among the phases, leading to the high flexibility of the blend (Zhang et al., 2020).

From Figure 5B, it can find that the addition of DCP alone increases the tensile strength of the blends, while the single addition of ADR significantly improves the elongation at break of the blends. When ADR and DCP are both introduced into the system, the tensile strength and elongation at break of the blend are further improved, which indicates that the two reactive additives play a good synergistic role in enhancing the tensile properties of the PLA/*i*-BIIRs blends. However, when the blend ratio was changed (Figure 5C), the tensile strength of the corresponding blends decreased with the increase of ionomer content, while the elongation at break increased slightly. Compared with AR-OH(30)+AD, the tensile strength of AR-OH(40)+AD was significantly reduced. Many studies have shown that the introduction of too much elastomer will inevitably lead to the reduction of the modulus and tensile strength of the blend system (Zhang et al., 2012; Lu et al., 2014). Therefore, the ionomer content of 30 wt% in the blend is the optimum formulation for the tensile properties.

**FIGURE 6:**
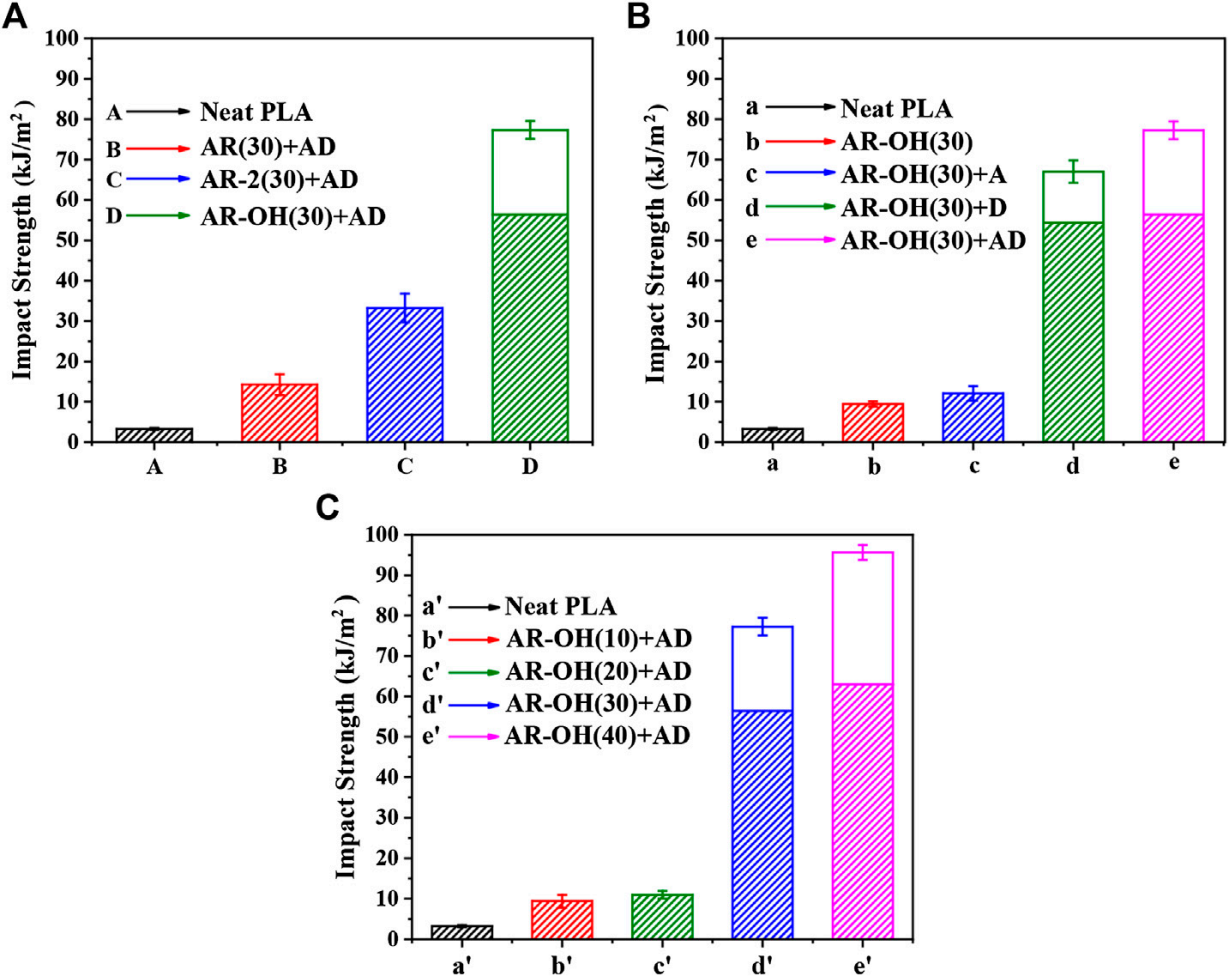
Impact strength of the neat PLA and the PLA/*i*-BIIRs blends: **(A)** neat PLA and PLA/*i*-BIIRs blends with ADR and DCP added and 30 wt% content of ionomer, **(B)** neat PLA and PLA/*i*-BIIR-OH blends with different addition of ADR and DCP, **(C)** neat PLA and PLA/*i*-BIIR-OH blends with different ionomer content.

Poor impact toughness is a key challenge for the industrial use of PLA. Figure 6 display the impact strength of the neat PLA and PLA/*i*-BIIRs blends. Compared with pure PLA, the impact strength of the PLA/*i*-BIIRs blends was remarkably improved to a high value. When the ionization-modified BIIR ionomer was used for reactive blending, the impact strength of the blends was significantly enhanced compared with AR(30)+AD blended with the pure BIIR. Among them, the impact strength of AR-OH(30)+AD blend reaches 77 kJ/m^2^, which is about 22 times higher than the impact strength of neat PLA (3.3 kJ/m^2^). However, it can be seen in Figure 6B that only adding ADR during the reactive blending has limited improvement in the impact strength of the blends, while the addition of DCP significantly improves the impact strength of the blends. This is consistent with the SEM results. The reactive blending enhances the interfacial adhesion of the blends, which contributes to the improvement of the impact strength of the blends. At the same time, the introduction of the reactive additives also reduces the size of the dispersed phase particles, which is beneficial to the shear yielding of the PLA matrix and the absorption of impact energy (Liu et al., 2014). According to the theory of Wu, in the blends of rubber-toughened brittle polymers, the relationship between the optimum rubber particle diameter (*d*_*o*_) and the entanglement density (*ν*_*e*_) of the brittle polymer is in accordance with the following equation (Wu, 1990; Wu, 1992).

$$logd_{o}=1.19-14.1\upsilon_{e}$$

Since the *ν*_*e*_ of PLA is in the range of 0.12–0.14 mmol/cc, the *d*_*o*_ of PLA can be calculated to be in the range of 0.1–0.3 μm (Joziasse et al., 1994). After the introduction of DCP, the particle size of the *i*-BIIR-OH is close to the *d*_*o*_ of PLA, especially the sample of AR-OH(30)+AD, so the reactive blending provides a good toughening effect. In the system, DCP has higher reactivity than ADR to obtain a higher content of reactive graft copolymers to achieve the compatibilization of the interphase of the blends, thereby significantly improving the compatibility and interfacial strength of the blends. Excellent interracial adhesion is a prerequisite for achieving the ideal toughening effect of the blend. The high impact toughness of the AR-OH(30)+AD blend is attributed to the combined compatibilizing effect from multiple intermolecular interactions between the ionomer and PLA matrix, the chain extension reaction of hydroxyl groups in ionomers and PLA end groups by ADR and the *in-situ* reactive graft initiated by DCP. Meanwhile, the elastomer content in the blend also has a huge influence on the impact properties of the material. It shows that the brittle-ductile transition of the material can be achieved only when the ionomer content reaches a certain value. Only when the ionomer content reaches 30%, the impact strength of the blend is significantly improved.

### 3.4 Impact Fracture Morphology

**FIGURE 7:**
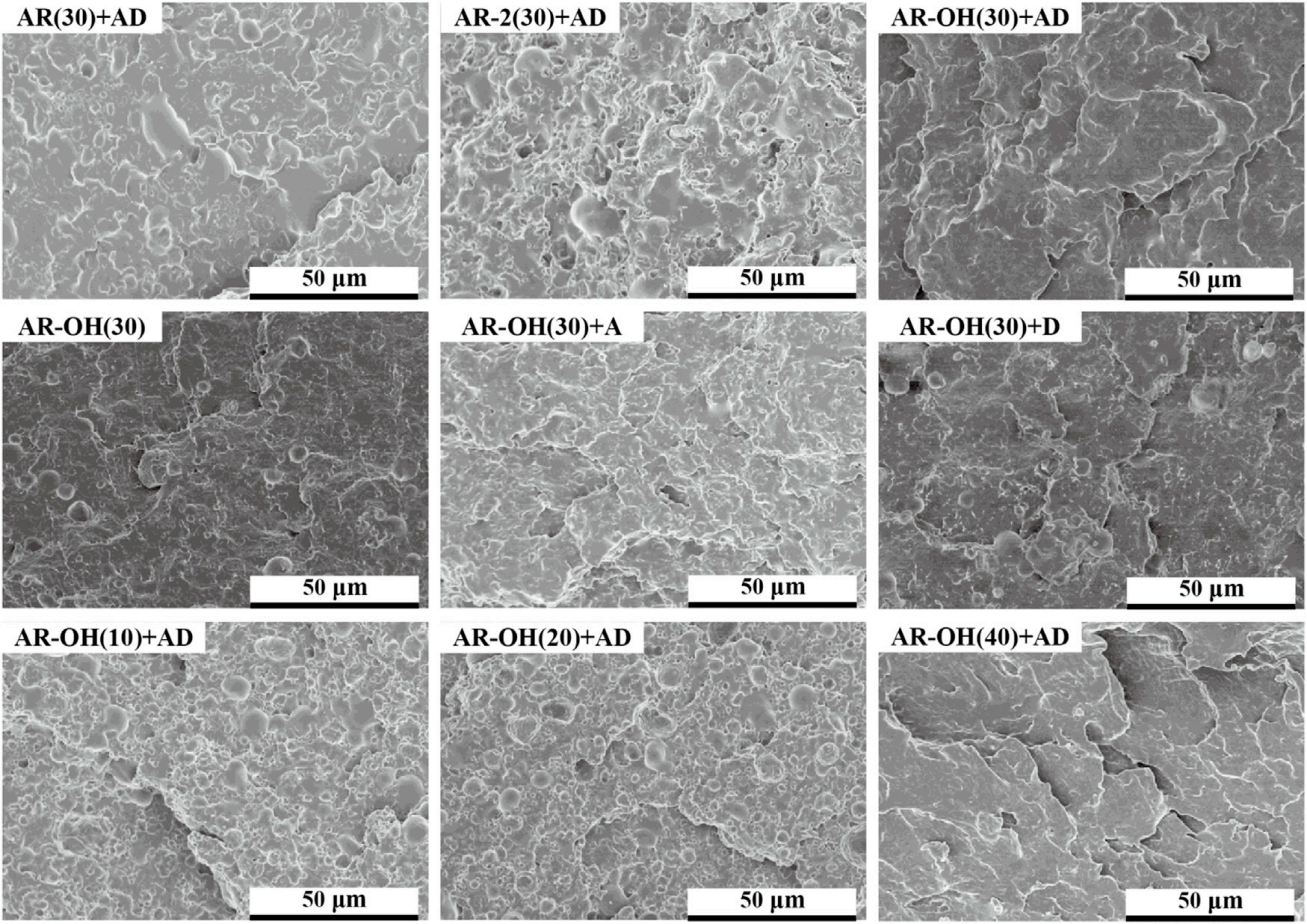
SEM images of the impact fracture surfaces of the PLA/*i*-BIIRs blends.

To further analyze the toughening mechanism of this reactive blending system, we observed the impact fracture morphologies of the PLA/*i*-BIIRs blends using SEM, and the results are shown in Figure 7. In the impact fracture morphologies of AR(30)+AD and AR-OH(30), the PLA matrix has no obvious yield deformation, and the overall section is relatively flat, showing the characteristics of brittle fracture. It indicates that using one strategy of reactive blending or the introduction of ionic groups alone cannot achieve the optimal toughening effect. In contrast, under the synergistic effect of the two strategies, the impact fracture morphologies of AR-OH(30)+D and AR-OH(30)+AD were rougher and showed larger interface fluctuations, which fully indicated that the PLA matrix had obvious yield deformation under the impact force. Owing to good interfacial compatibility, the peeling or self-cavitation of the elastomer phase at the interface can effectively induce the yield deformation of the surrounding PLA matrix, leading to absorbing a large amount of energy when the impact occurs. Accordingly, it will substantially increase the impact strength of the blend. At the same time, the reactive blending improves the compatibility of the ionomer with the PLA matrix and increases the interfacial strength of the blends, which reduces the generation and growth of micro-cracks at the phase interface and promotes the yield deformation of the PLA matrix. In summary, the good compatibility of the blends is the key factor for the significant improvement of the impact strength of the blends, and the synergistic effect of reactive blending and multiple intermolecular interactions plays an important role.

## 4 Conclusion

In this work, ultra-tough sustainable PLA-based blends were successfully prepared by reactive blending with the BIIR-based elastomeric ionomers using ADR and DCP as reactive agents. The introduction of OH-functional ionic groups and reactive agents played a good synergistic effect on reactive compatibilization, which effectively improved the compatibility and mechanical properties. The interfacial *in-situ* grafting reaction and cross-coupling induced by reactive blending promoted the uniform dispersion of the ionomers phase with strong interfacial adhesion. The optimum formulation AR-OH(30)+AD shows the highest elongation at break of 286% and impact strength reached 77 kJ/m^2^. In summary, we demonstrates that the combining multiple intermolecular interactions and reactive blending is a very effective and facile strategy for toughening PLA blend, which has great potential to be used to toughen other polymer blending systems.

## Data Availability

The original contributions presented in the study are included in the article/Supplementary Material, further inquiries can be directed to the corresponding authors.
